# UsbVisdaNet: User Behavior Visual Distillation and Attention Network for Multimodal Sentiment Classification

**DOI:** 10.3390/s23104829

**Published:** 2023-05-17

**Authors:** Shangwu Hou, Gulanbaier Tuerhong, Mairidan Wushouer

**Affiliations:** Xinjiang Multilingual Information Technology Laboratory, Xinjiang Multilingual Information Technology Research Center, College of Information Science and Engineering, Xinjiang University, Urumqi 830017, China

**Keywords:** multimodal sentiment classification, user behavior attention, visual attention

## Abstract

In sentiment analysis, biased user reviews can have a detrimental impact on a company’s evaluation. Therefore, identifying such users can be highly beneficial as their reviews are not based on reality but on their characteristics rooted in their psychology. Furthermore, biased users may be seen as instigators of other prejudiced information on social media. Thus, proposing a method to help detect polarized opinions in product reviews would offer significant advantages. This paper proposes a new method for sentiment classification of multimodal data, which is called UsbVisdaNet (User Behavior Visual Distillation and Attention Network). The method aims to identify biased user reviews by analyzing their psychological behaviors. It can identify both positive and negative users and improves sentiment classification results that may be skewed due to subjective biases in user opinions by leveraging user behavior information. Through ablation and comparison experiments, the effectiveness of UsbVisdaNet is demonstrated, achieving superior sentiment classification performance on the Yelp multimodal dataset. Our research pioneers the integration of user behavior features, text features, and image features at multiple hierarchical levels within this domain.

## 1. Introduction

We have likely all encountered people who are extremely positive or negative when evaluating products. However, these opinions can significantly impact a product’s overall evaluation due to software systems calculating sentiment averages. This issue has led to the emergence of affective computing, which aims to understand human emotions by monitoring facial, vocal, and bodily behaviors (Calabrese & Cannataro, 2016) [1]. Affective computing is associated with emotions, originates from emotions, or influences emotions (Lisetti, 1998) [2], while sentiment analysis attempts to discover user emotions through text data. Although these two research disciplines use different methods to determine user emotions, they share a common goal: identifying user emotions. As a result, both types of research may recognize user psychological behaviors.

In this study, we adopt a key assumption of affective computing: human behavior and emotions are not entirely dependent on rules and regulations. This implies that users’ emotions towards a business are not always based on logic or entirely emotional, and users may sometimes hold extreme biases, located at the poles of opinion polarity. Some users are consistently negative, while others are consistently positive. This means that some people are inherently pessimistic, frequently expressing negative opinions about restaurants, movies, or other aspects of life. In contrast, others are inherently optimistic, often offering positive reviews about various things. Additionally, user behavior, such as socializing, experience, and lifespan on social networks, significantly influences user reviews on social networks.

In this study, we aim to demonstrate that users’ psychological behaviors directly impact understanding user attitudes and aid in the sentiment classification of user review text content. Identifying biased users in product reviews is crucial for two main reasons. First, in sentiment analysis, biased user reviews may adversely affect a business’s evaluation. Identifying such users is highly beneficial since their reviews are based on their characteristics rooted in their psychology rather than reality. Second, biased users may also be perceived as instigators of other prejudiced information on social media. Therefore, proposing a method that helps detect polarized opinions in product reviews offers significant advantages. To our knowledge, no other research has been conducted in the multimodal sentiment analysis domain focusing on user behavior. Consequently, this study’s results will benefit society as a whole.

In this research, we first analyze the role of user characteristics and behaviors in review text, then demonstrate how to extract user attitudes based on their psychological behaviors. We assume that more sociable individuals are more positive than isolated ones, and those with more experience on e-commerce platforms are more positive than those without experience. We consider features such as sociability (e.g., number of friends) and experience (e.g., user lifespan) as user characteristics. To achieve our research objectives, we use the Yelp multimodal dataset. We first extract important features representing user behavior from these datasets, then calculate user sentiment within the dataset. We then elaborate on the relationship between user sentiment and user behavior. The proposed model employs a sentiment classification approach based on user behavior attention to predict biased user presence based on their psychological behavior, thereby improving sentiment classification performance. The remainder of the content will detail the proposed model, dataset, and experiments, followed by a discussion of the experimental results.

## 2. Related Work

The feature fusion process based on attention mechanisms relies on generating dynamic scalar weights using a small attention model at each step. These weights are then used to weight a set of vectors, resulting in attended vectors. Typically, multiple sets of dynamic weights are created using multiple attention heads, which can combine the results from each head to preserve more information. When applying attention mechanisms to images, different feature vectors associated with different regions are assigned different weights to create an attended image vector, as seen in the work of Zhang et al. [3]. In contrast, Cao et al. [4] adopt an asymmetric attention framework to generate attended image and textual feature vectors, while Xu et al. [5] use a dual attention network (DAN) to simultaneously predict the attention distribution of both the image and the text. Unlike collaborative attention, where an asymmetric attention framework is used to generate attended feature vectors, memory vectors can be repeatedly modified at each inference level using a repeated DAN structure. Xu et al. [6] propose an alternating collaborative attention mechanism, which generates an attended image vector based on textual features and then generates an attended text vector based on the attended image vector. In addition, Yang et al. [7] and Zhang et al. [8] propose multi-head attention models to define the correlation between image and text content. Gated multimodal fusion is considered a different type of attention mechanism, as it uses gates to allocate weights to visual and textual elements. The weighted sum of visual and textual feature vectors can be computed based on scalar weights specific to dimensions, which are automatically created using the gating process. These representations can then be used for multimodal sentiment classification, as described in the work of Huang et al. [9] and Arevalo et al. [10].

Jin and Zafarani (2018) [11] have demonstrated the possibility of predicting user sentiment in social networks by analyzing content. Their study used structural properties at different levels, such as the ego, triad, community, and network levels, to predict sentiment. Tan et al. (2013) [12] also explored the concept of social relationships to improve sentiment analysis at the user level on Twitter. Similarly, Yang and Eisenstein (2017) [13] presented a method to overcome language variation in sentiment analysis by incorporating social attention, while Yang et al. (2016) [14] leveraged the sociological theory of homophily to address the ambiguity problem in Twitter text. Tang and Liu (2015) [15] introduced a model called the user product neural network to extract user and product information and improve sentiment classification. Gui et al. (2017) [16] also combined user or product information with text information to train a sentiment classifier. Gong and Wang (2018) [17] proposed a novel method based on the self-consistency theory to discover user behavior in social media. Zou et al. (2018) [18] developed a model that utilized social and topic content to identify user sentiment. Fornacciari et al. (2015) [19] combined social network structure with sentiment analysis to demonstrate how sentiment analysis could produce incorrect results depending on the network topology. In the context of this study, the number of friends in an online social network (OSN) is used as a predictor of positive attitude based on the social psychological characteristic of socialization. Rubin and Bowker (2018) [20] suggest that the number of friends is one of the main criteria for being socialized. Asocial individuals (i.e., those with very few friends) are assumed to have a negative attitude in the OSN context. Friendship is a significant factor for life satisfaction, and positivity in an individual can alter the attitude of their friends. Allport (1935) [21] defines attitude as a “mental and neural state of readiness, organized through experience”, and Fazio and Zanna (1981) [22] demonstrated through a series of studies that direct behavioral experience with an attitude object is a better predictor of subsequent behavior. Long-term membership in OSNs can enhance users’ experience, thereby affecting their attitude. The relationship between sentiment and user behavior in OSN datasets is the focus of this study, and it aims to pave the way for psychologists to provide a theory about this relationship. It is important to note that any evidence of causation would need to be provided by a psychologist as this study can only demonstrate the existence of a relationship between the above-mentioned factors without proving causation.

Recently, Wang et al. [23] proposed a method called “Deepvid” for the visual interpretation and diagnosis of image classifiers using knowledge distillation techniques. This method uses an auxiliary model to learn the decision process of the main classifier and maps these decisions into visualized heatmaps. These heatmaps show which parts of the input image the main classifier pays more attention to and which parts are ignored. Through this approach, users can better understand and analyze the behavior of image classifiers, and developers can improve the performance and accuracy of the model. Ma et al. [24] proposed an adaptive localized decision variable analysis approach for solving large-scale multiobjective and many-objective optimization problems. The proposed approach identifies important decision variables that contribute to the objective functions by partitioning the decision variables into small subsets and analyzing them locally. The authors evaluated their approach to several benchmark problems and compared it with other state-of-the-art methods. The results show that their approach outperforms existing methods in terms of accuracy and efficiency. Truong et al. [25] proposed a model called “Vistanet” for analyzing emotions from multimodal data. The model uses a visual aspect attention network to perform sentiment analysis on image and video data and employs a self-attention mechanism to consider interactions between text and visual data. The experimental results demonstrate that the Vistanet model achieves better performance than other methods on multiple benchmark datasets. Zhang et al. [26] presented various methods for emotion recognition using multiple data types, including text, audio, images, and videos. The article then delved into the application of machine learning in emotion recognition, including different learning methods such as supervised learning, unsupervised learning, and semi-supervised learning, and compared and analyzed the advantages and disadvantages of different methods. Finally, the article discussed and prospected the challenges and future research directions in the field of emotion recognition. Zhao et al. [27] provide a comprehensive review of emotion recognition methods using multiple modalities, including facial expression, speech, physiological signals, and text. The authors discuss the fundamental challenges and methodologies of multi-modal emotion recognition, including feature extraction, fusion techniques, and machine learning models. The paper also covers the recent advances in deep learning-based methods and their applications in multi-modal emotion recognition. Finally, the authors provide insights into future research directions and potential applications in real-world scenarios.

## 3. Definition

### 3.1. User Relationship Network

Each User Relationship Network (URN) can be represented as G(U,C,T), where *U* is a set of user IDs, *C* is a set of triples in the form of $\left(U_{i},U_{j},t\right),i,j\in \left(0,K\right]$ where $(U_{i},U_{j}\in U),\;t\in T,\;T$ is a collection of timestamps when users $u_{i}$ and $u_{j}$ communicate with each other, and *T* can typically be regarded as the time when a connection is established between the two users. *K* represents the total number of users in the user set *U*. According to the aforementioned definition, the User Relationship Network (URN) is a dynamic domain where network behaviors change over time.

### 3.2. Sentiment

In this study, we measure the sentiment of URN users by calculating the sentiment scores for each review text (in Yelp). For each user $U_{k},k\in \left(0,K\right]$ who posts reviews on certain topics, a sentiment score feature vector is calculated and represented as $S_{U_{k}}=\left\{s_{k,1},s_{k,2},\ldots ,s_{k,N}\right\}$, where $s_{k,n},n\in \left(0,N\right]$ denotes the sentiment score of the *n*-th review posted by user $U_{k}$, and *N* represents the total number of reviews posted by user $U_{k}$. Each user in the URN has a certain number of reviews. $R_{U_{k}}=\left\{r_{k,1},r_{k,2},\ldots ,r_{k,N}\right\}$ represents the set of all reviews posted by user $U_{k}$, as described in Equation (1):

(1) $$S_{U_{k}}=Sentiment\left(R_{U_{k}}\right)$$

In Equation (1), Sentiment is a function that calculates the sentiment score of the review text, and the sentiment score can be either positive or negative.

### 3.3. Attitude

In this study, for each user $U_{k}$, a value is assigned to their attitude based on the degree of liking or disliking (i.e., sentiment score) expressed in their review text. The overall user attitude *A* is formed by the sentiment scores of each user $U_{k}$ and can be represented as $A=\{a_{1},a_{2},\ldots ,a_{K}\}$ in the URN, where each $a_{k},k\in \left(0,K\right]$ is associated with each user ($U_{k}$) in the URN. Equation (2) is used to calculate the attitude of each user. The sum of user sentiment scores divided by the number of reviews is considered as the user attitude, as shown below:

(2) $$a_{k}=\sum_{n=1}^{N}s_{k,n}$$

In Equation (2), *N* represents the total number of reviews posted by user $U_{k}$; $a_{k}$ is the attitude of user $U_{k}$; and $s_{k,n}$ is the sentiment score of the *n*-th review text by user $U_{k}$.

If the user’s attitude score is positive, we define user $U_{k}$ as a user with a positive attitude. If the user’s attitude score is negative, we define user $U_{k}$ as a user with a negative attitude. We represent the attitude of user $U_{k}$ towards a particular product with $UA_{k}$, as shown in Equation (3):

(3) $$UA_{k}=\left\{\begin{array}{c} positive,\;\;a_{k}>0\\ negative,\;\;a_{k}<0 \end{array}\right.$$

### 3.4. Biased Users

In this study, our definition of bias is related to our definition of attitude. This means that we consider biased individuals to have excessively positive or excessively negative attitudes in most cases. Furthermore, since this is a novel topic in the field of sentiment analysis, the definition of bias in this context is not precise. Therefore, in this study, we consider a statistical criterion based on the number of sentiment values so that multiple definitions can be included in the future. To achieve this, we obtain the sentiment distribution, meaning we see how many sentiment values are normal, and then we use standard deviation to determine the abnormal amount of sentiment. For example, to calculate the user sentiment distribution, we first calculate the user sentiment mean (μ) and the user sentiment standard deviation (σ), and then calculate the sentiment distribution concerning (μ, σ).

(4) $$\mu =\frac{1}{K}\sum_{k=1}^{K}a_{k}$$

where $a_{k}$ is the attitude of the *k*-th user in the user set, and *K* is the total number of users.

(5) $$\sigma =\sqrt{\frac{1}{K-1}\sum_{k=1}^{K}{\left(a_{k}-\mu \right)}^{2}}$$

where $a_{k}$ is the attitude of the *k*-th user in the user set, μ is the mean of user attitudes, and *K* is the total number of users.

Now, based on different numbers of standard deviations, biased users can be classified into different groups. For example, sentiment values between μ−xσ and μ+xσ can be considered as normal users, while values less than μ−xσ and greater than μ+xσ can be considered as biased users. In this study, we classify biased users $BU_{k}$ as follows based on Equation (6):

(6) $$BU_{k}=\left\{\begin{array}{c} overly\;positive,\;\;a_{k}\;\geq \;\mu +x\sigma \\ overly\;negative,\;\;a_{k}\;\leq \;\mu -x\sigma \end{array}\right.$$

This means that users with $\mu -x\sigma <BU_{k}<\mu +x\sigma$ are normal users, while others are biased users. Here, *x* represents the degree of bias value; the closer *x* is to 0, the higher the degree of bias $BU_{k}$, and the more extreme the reviews of user $U_{k}$ are.

## 4. Theoretical Foreshadowing

In order to analyze the role of user features in review texts, we first extracted available user features from the dataset. These features include the number of friends, number of fans, age of the user’s account, and the number of reviews they have posted. Next, we identified the relationships between these attributes and biased users and used them as a basis for a novel sentiment analysis model based on user behavior. To calculate the sentiment score $s_{k,n}$ of user review texts, we used the RSentiment package in the R programming language.

(7) $$s_{k,n}=RSentiment\left(r_{k,n}\right),\;\;\;k\in \left(0,K\right],n\in \left(0,N\right]$$

(8) $$S_{U_{k}}=\left\{s_{k,n}\;|\;n\in \left(0,N\right]\right\}$$

One of the key parts of this study is feature selection (i.e., selecting user features—psychological behavior features). In this study, we used the user’s text to extract these features. One of the primary tools used is the correlation matrix, which displays the degree of association between features. In this study, we attempted to find the degree of association between user features $E_{U_{k}}$ and user sentiment value features $S_{U_{k}}$. The correlation coefficient represents the strength of the association. There are two main types of correlation coefficients: Spearman and Pearson (Schober et al., 2018) [28]. We used the Spearman rank correlation coefficient in this study, as the Yelp dataset contains sentiment (emotional) variables, which are of an ordinal data type. The Spearman rank correlation coefficient is suitable for this data type, so we used it to measure the degree of association between the aforementioned user features and sentiment scores.

### Spearman Rank Correlation Coefficient

The correlation coefficient cr can be calculated as follows: Suppose user $U_{k}$ has two variables: user features $E_{U_{k}}$ and user sentiment value features $S_{U_{k}}$, each containing values $e_{k,1},\;e_{k,2},\;\ldots ,\;e_{k,N}$ and $s_{k,1},\;s_{k,2},\;\ldots ,\;s_{k,N}$. Let the average of $E_{U_{k}}$ be $\bar{E_{U_{k}}}$ and the average of $S_{U_{k}}$ be $\bar{S_{U_{k}}}$. Then, cr is:

(9) $$cr=\frac{\sum \left(e_{k,n}-\bar{E_{U_{k}}}\right)\left(s_{k,n}-\bar{S_{U_{k}}}\right)}{\sqrt{{\left(e_{k,n}-\bar{E_{U_{k}}}\right)}^{2}{\left(s_{k,n}-\bar{S_{U_{k}}}\right)}^{2}}}$$

**Figure 1:**
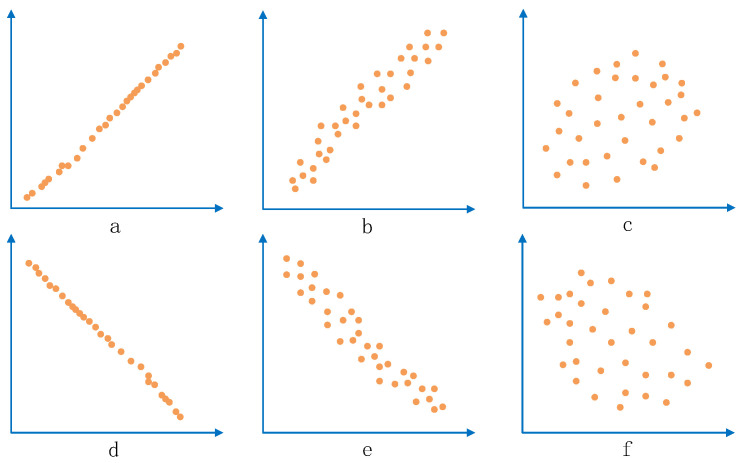
Correlation coefficient cr between two variables. ((**a**) shows a strong positive correlation, (**b**) shows a weak positive correlation, (**c**) shows a very weak (negligible) positive correlation, (**d**) shows a strong negative correlation, (**e**) shows a weak negative correlation, and (**f**) shows a very weak (negligible) negative correlation.)

The correlation coefficient cr is a summary measure that describes the degree of statistical relationship between two variables. The correlation coefficient ranges between −1 and +1. When the correlation coefficient takes values of +1 or −1, the association between the two variables is stronger, indicating a definite relationship. A correlation coefficient close to +1 indicates a positive relationship between the two variables, while one close to −1 indicates a negative relationship. A correlation coefficient of 0 indicates no association between the two variables. Figure 1 shows the cr values for the degree of correlation between different sample values. In Figure 1, (a) and (d) shows a strong correlation, (b) and (e) shows a weak correlation, (c) and (f) shows a very weak (negligible) correlation.

## 5. UsbVisdaNet

**Figure 2:**
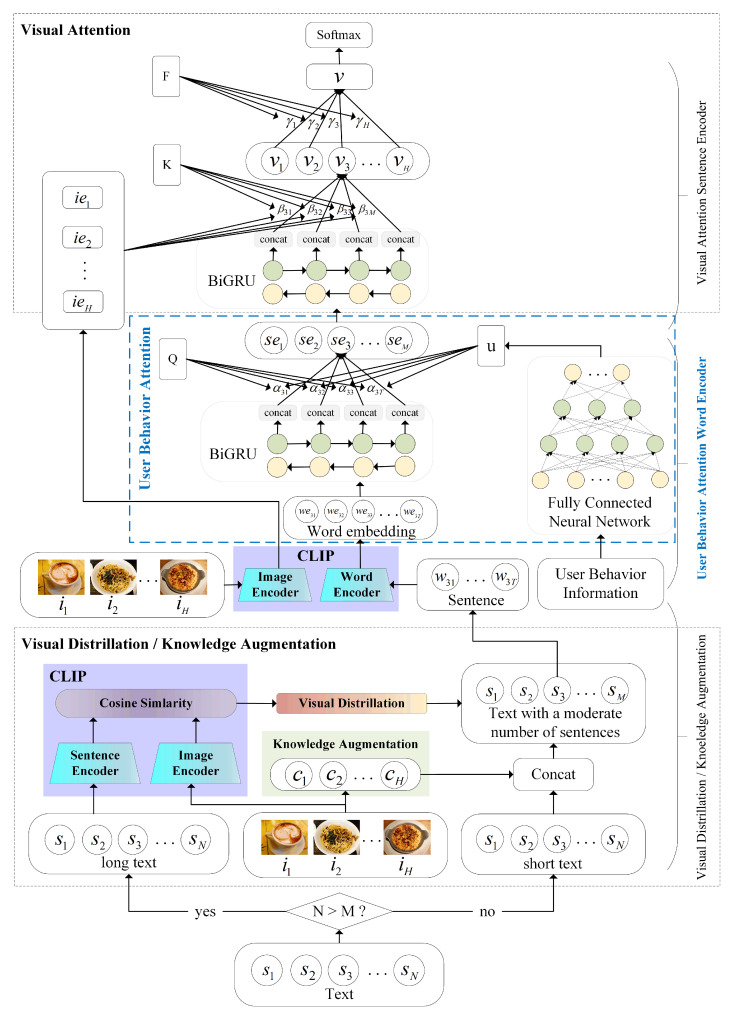
User Behavior Visual Distillation and Attention Network Model Structure.

Later, in Section 6.3, we demonstrate through experiments that there is an association between user features and sentiment values in the dataset. Therefore, in this section, we make use of the aforementioned user feature theory and incorporate the user features into our previously proposed VisdaNet [29] model. We improve the “word encoder with word attention” layer in the VisdaNet [29] model and propose the “word encoder with user behavior attention” in this section. We call the improved model the User Behavior Visual Distillation and Attention Network (UsbVisdaNet), as shown in Figure 2.

**Problem Definition**. We have a set of user comments, denoted as *R*, where each comment r∈R is represented as {(txt,uf,imgs),y}. Here, txt is the textual component of the comment, uf represents user behavior features, imgs is the visual component of the comment, and *y* represents the sentiment label of the comment. The textual component txt is a sequence of *N* sentences $s_{n}$, where txt can be expressed as $\left\{s_{n}|n\in \left[1,N\right]\right\}$, and each sentence $s_{n}$ comprises *T* words $w_{n,t}$ such that $s_{n}$ can be expressed as $\left\{w_{n,t}|t\in \left[1,T\right]\right\}$. The visual component imgs includes a series of *H* images $i_{h}$ with their respective image descriptions $c_{h}$, such that imgs can be represented as $\left\{\left(i_{h},c_{h}\right)|h\in \left[1,H\right]\right\}$. The objective of multimodal sentiment classification is to train a classification function *f* using labeled training data *R*, which can predict the sentiment polarity of previously unseen multimodal samples containing both textual and visual components, i.e., f(txt,uf,imgs)=y.

Our UsbVisdaNet model is entirely based on improvements to the VisdaNet [29] model. The architecture of UsbVisdaNet consists of four hierarchical layers, as illustrated in Figure 2. The proposed model, UsbVisdaNet, is comprised of four layers, each with a specific function. The first layer, situated at the bottom, functions as a visual distillation and knowledge augmentation layer, addressing the challenge of information control during multimodal fusion in product reviews. The visual distillation module compresses lengthy texts to eliminate noise and improve the quality of the original modalities. In contrast, the knowledge augmentation module incorporates image descriptions and short text to enrich short text information, compensating for the limited information present in short texts. The second layer is the word encoding layer with user behavior attention, which converts features from the word level to the sentence level. The third layer is the sentence encoding layer, which addresses the challenge of integrating multiple images with a single text in the context of product reviews by incorporating visual attention mechanisms based on CLIP [30]. This approach is used to transform sentence features into features for the entire comment. The top layer is a classification layer used to calculate the sentiment labels of user reviews. To avoid redundancy, we will not reiterate the parts that are the same as VisdaNet in this section; instead, we will focus on the differences and improvements compared to VisdaNet in the remaining parts of this section. Next, we will offer a more elaborate explanation of the design specifics for every layer.

### 5.1. Knowledge Augmentation/Visual Distillation

In this section, we follow the approach utilized in VisdaNet [29] to differentiate between short and long texts based on the number of sentences, denoted as *N*. Texts with *N* less than a hyperparameter *M* are classified as short texts and processed using the knowledge augmentation (KA) algorithm [29] to augment their information content. Conversely, texts with *N* greater than *M* are classified as long texts and processed using the visual distillation (VD) algorithm. *M* is used as a hyperparameter and its value is adjusted during training.

#### 5.1.1. Knowledge Augmentation

For short texts, we utilize an advanced image captioning model DLCT [31] to generate image descriptions $c_{h}$ for the accompanying images $i_{h}$ in the review, thereby augmenting the text with additional knowledge (KA) [29]. After applying the KA algorithm, we can augment the number of sentences in a short review from *N* to *M*, resulting in a sentence set $S^{M}$:

(10) $$c_{h}=DLCT\left(i_{h}\right),h\in \left[1,H\right]$$

(11) $$S^{M}=KA\left(s_{n},c_{h},M\right),n\in \left[1,N\right],h\in \left[1,H\right]$$

#### 5.1.2. Visual Distillation

**Figure 3:**
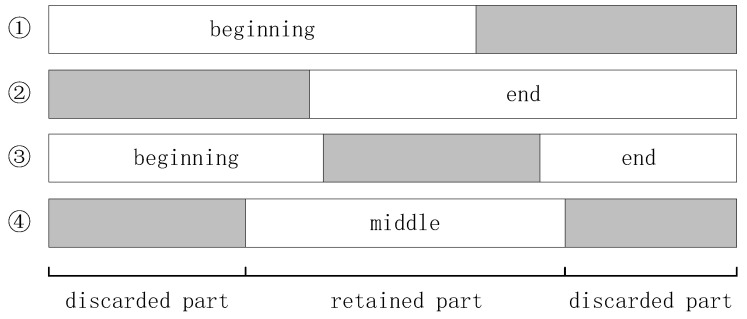
The 4 traditional method of text truncation.

Due to model limitations, hardware constraints, and computing power, models often can only handle text content of limited lengths during training. For example, the BERT [32] model imposes a maximum character length limit of 512 for input data. Prior works on multimodal sentiment classification, such as Yang et al. [33] and Truong et al. [25], have utilized a direct truncation strategy during training. This strategy involves retaining the first part of a text and discarding the rest for texts exceeding a certain length threshold *M*, as depicted in Figure 3. However, all the traditional truncation methods depicted in Figure 3 have flaws in this application. If long texts are processed using these traditional truncation strategies, important sentiment information contained in the discarded part would be lost, which might not necessarily be unimportant. These traditional truncation strategies are clearly unreasonable. Therefore, we propose a visual distillation module, based on the KD algorithm [29] from VisdaNet [29], which we renamed as the VD algorithm, to improve upon the traditional truncation strategies.

After applying the *VD* algorithm, we can reduce the number of sentences in a long review from *N* to *M*, resulting in a sentence set $S^{M}$:

(12) $$S^{M}=VD\left(sim\left(T\left(s_{n}\right),I\left(i_{h}\right)\right),M\right),n\in \left[1,N\right],h\in \left[1,H\right]$$

In Equation (12), T represents the CLIP text encoder, I represents the CLIP image encoder, and sim denotes the calculation of cosine similarity.

### 5.2. User Behavior Attention Mechanism

Not all user features are relevant to sentiment, so we need a model criterion based on user features to filter them out. Additionally, to increase the model’s complexity and expressiveness, we feed the extracted salient features into a two-layer fully connected neural network to extract higher-level features. Then, we incorporate the user features into the word attention mechanism to allow the model to focus more on the text features with important user features.

#### 5.2.1. User Feature Extractor

We propose a model criterion based on user features. Section 6.3 demonstrates that certain features of users, such as “Friend Count (FN)”, “Network Age (NA)”, and “Review Count (RN)”, are crucial in forming user attitude $UA_{k}$. Firstly, we normalize these user feature values $uf,uf=uf_{1},uf_{2},\ldots ,uf_{M}$ using Equation (13):

(13) $$norm\_uf_{m}=\frac{uf_{i}-min\left(uf\right)}{max(uf)-min(uf)},m\in \left(0,M\right]$$

(14) $$norm\_uf=\{norm\_uf_{1},norm\_uf_{2},\ldots ,norm\_uf_{M}\}$$

Next, based on the extracted features, we define a criterion based on normalized values and the role of each feature in user attitude $UA_{k}$. Significant features sf can be derived from the values of the correlation coefficient, principal component analysis, and factor analysis. As shown later in Section 6.3, we use a correlation matrix to extract significant features.

(15) sf=correlation_matrix(norm_uf)

(16) $$sf=\{sf_{1},sf_{2},\ldots ,sf_{L}\},L\leq M$$

Based on the extracted significant features, to increase the model’s complexity and expressiveness, we employ two fully connected layers to better extract user information features. The first fully connected layer maps the input data to a hidden layer feature space, where each neuron corresponds to a learned feature. The second fully connected layer further combines and transforms the features in the hidden layer to extract higher-level features that can better describe users’ behavior and emotional states. By using two fully connected layers, the model can learn more complex user behavior features *u*, which can better distinguish different users and emotional states. Moreover, a multi-layer fully connected network can also be trained end-to-end using the backpropagation algorithm to minimize the loss function and improve the model’s prediction accuracy.

(17) $$u=\sigma (w_{2}f\left(w_{1}\;sf+b_{1}\right)+b_{2})$$

In Equation (17), sf represents the input user’s significant information, $w_{1}$ and $w_{2}$ represent the weights in the two fully connected layers, $b_{1}$ and $b_{2}$ represent the biases in the two fully connected layers, σ denotes the sigmoid activation function, and *u* represents the output user behavior features.

#### 5.2.2. Word Encoder with User Behavior Attention

We split $S^{M}$ into sentences $s_{m}$, and further split sentences $s_{m}$ into words $w_{m,t}$. We encode the words $w_{m,t}$ using the CLIP word encoder to obtain word embeddings $we_{m,t}$:

(18) $$we_{m,t}=T\left(w_{m,t}\right),t\in \left[1,T\right]$$

Then, we utilize BiGRU to obtain a new feature representation $\epsilon_{m,t}$ for the review, addressing the challenges of word order and word ambiguity:

(19) $$\epsilon_{m,t}=\left[\vec{GRU}\left(we_{m,t}\right);\overset{\leftarrow }{GRU}\left(we_{m,t^{\prime }}\right)\right],t\in \left[1,T\right],t^{\prime }\in \left[T,1\right]$$

In our UsbVisdaNet, we improve the “Word Encoder with Word Attention” in VisdaNet [29], as shown in Equation (20):

(20) $$q_{m,t}=tanh\left(u^{T}W_{q}\epsilon_{m,t}+b_{q}\right)$$

We add the user feature *u* to Equation (20) to introduce user features into the soft attention mechanism, allowing the model to pay more attention to text features with important user features. In this process, the weight of user features is determined by the soft attention mechanism. In this way, the model can better utilize the relationship between user features and text features to improve the accuracy of sentiment classification. At the same time, incorporating *u* ensures the interpretability of the model, and the attention weights can be analyzed to understand the model’s focus on different user features and text features.

Subsequently, during the training phase, we utilize the user behavior word attention mechanism to acquire the user behavior attention weights denoted as *Q*, which are further normalized to derive $\alpha_{m,t}$:

(21) $$\alpha_{m,t}=\frac{exp(Q^{T}q_{m,t})}{\sum_{t}^{}exp\left(Q^{T}q_{m,t}\right)}$$

Ultimately, we derive the sentence embedding $se_{m}$ by obtaining the weighted sum of all word representations $\epsilon_{m,t}$, with the normalized attention weights $\alpha_{m,t}$ used as the weights in the summation.

(22) $$se_{m}=\sum_{t}^{}\alpha_{m,t}\epsilon_{m,t}$$

### 5.3. Sentence Encoder with Visual Attention

We used the visual aspect attention mechanism in VistaNet [25], which consists of two layers: sentence-level visual attention and document-level attention. We will provide a more detailed explanation of this below.

We utilize BiGRU to obtain a new feature representation $\epsilon_{m}$ for the review, addressing the issue of sentence order:

(23) $$\epsilon_{m}=\left[\vec{GRU}\left(se_{m}\right);\overset{\leftarrow }{GRU}\left(se_{m^{\prime }}\right)\right],m\in \left[1,M\right],m^{\prime }\in \left[M,1\right]$$

We encode the image $i_{h}$ using the CLIP image encoder I to obtain the image embedding $ie_{h}$:

(24) $$ie_{h}=I\left(i_{h}\right),h\in \left[1,H\right]$$

In the training process, the model learns the sentence-level visual attention weight *K* of the sentence representation $\epsilon_{m}$ with respect to each image embedding $ie_{h}$ and normalizes *K* to obtain $\beta_{h,m}$. Finally, the sentence embedding $\epsilon_{1},\epsilon_{2},\ldots ,\epsilon_{m},\ldots ,\epsilon_{M}$ are aggregated with respect to each image $i_{h}$ into a comment representation $v_{h}$ using the normalized attention weight $\beta_{h,m}$:

(25) $$d_{h}=tanh\left(W_{d}ie_{h}+b_{d}\right)$$

(26) $$g_{m}=tanh\left(W_{g}\epsilon_{m}+b_{g}\right)$$

(27) $$k_{h,m}=d_{h}⊙g_{m}+g_{m}$$

(28) $$\beta_{h,m}=\frac{exp(K^{T}k_{h,m})}{\sum_{m}^{}exp\left(K^{T}k_{h,m}\right)}$$

(29) $$v_{h}=\sum_{m}^{}\beta_{h,m}\epsilon_{m}$$

Since there are multiple images and each image provides different guidance to the text, we need to learn the document-level attention weight *F* of the text with respect to each image during training and normalize *F* to obtain $\gamma_{h}$. Finally, the comment representations $v_{h}$ for multiple images are aggregated into the final comment representation *v* using the normalized document-level attention weight $\gamma_{h}$.

(30) $$f_{h}=tanh\left(W_{f}v_{h}+b_{f}\right)$$

(31) $$\gamma_{h}=\frac{exp(F^{T}f_{h})}{\sum_{h}^{}exp\left(F^{T}f_{h}\right)}$$

(32) $$v=\sum_{h}^{}\gamma_{h}v_{h}$$

### 5.4. Sentiment Classification

After collecting the high-level representation *v* of the comment *r*, we apply it as a feature to a softmax-based sentiment classifier at the top layer, which creates a probability distribution over classes rho. The model is then supervised-trained by reducing the sentiment classification’s cross-entropy loss.

(33) $$\rho =softmax(W_{r}v+b_{r})$$

(34) $$loss=-\sum_{v}^{}log\;\rho_{v,l^{T}}$$

In the Equation (34), $l^{T}$ represents the true label of the comment *v*.

## 6. Experiments and Analysis

To calculate sentiment values, this study used the sentiment package in the R programming language. Specifically, we employed the “sentiment_by” function of the RSentiment package. The output of this function includes the sentiment value “avg_sentiment”, which displays the sentiment (polarity) score. The Python 3.6 language was used to write all neural network code on an Ubuntu 18.04.9 operating system. TensorFlow 1.14.0 was the deep learning framework utilized. To expedite the training process, an Intel Core i9-9900K CPU @ 3.6 GHz × 16 and a GeForce RTX 3090 GPU were employed.

### 6.1. Dataset

The Yelp restaurant review dataset was utilized in the experiments, the dataset used in this study was collected from the Yelp restaurant review website and consists of pairs of text and images. The reviews were written for restaurants located in five cities across the United States. The textual information comprises the review’s body, tags, business information, and user information. The user information is presented in Table 1. The dataset provides rich user information, such as user_review_count (the number of reviews the user wrote), yelping_since (used to compute the user’s “age” on the platform), the number of friends (fans), and the average rating for all reviews (average_stars). The reviews are typically lengthy and contain many sentences. The majority of the reviews in the dataset are associated with three or more images, and the polarity of the sentiment expressed in each review is determined by the corresponding rating given by the reviewer, with higher ratings indicating greater user satisfaction with the product (restaurant or dish). The dataset is evenly distributed among the five categories, with a total of 44,305 samples. The samples are split into training, validation, and test sets in an 8:0.5:1.5 ratio. The test set is divided into five subsets based on the restaurants’ locations, namely Boston (BO), Chicago (CH), Los Angeles (LA), New York (NY), and San Francisco (SF). The dataset’s statistical information is presented in Table 2.

### 6.2. Experimental Setup

In the experiments for our model, we set the bias degree value *x* to 3. We use the Adam [34] algorithm for gradient-based optimization during the training process, with a learning rate of 0.0001. Table 3 lists the other hyperparameters for the experiments.

Table 4 illustrates the complexity, processing speed, and classification performance of the UsbVisdaNet model with respect to the hyperparameter *M* (number of sentences per comment). It shows that the impact of the hyperparameter *M*, which corresponds to the maximum number of sentences per comment, on the classification performance of our model. And it shows that the training time increases as *M* increases. However, the optimal classification performance is not achieved when *M* is too small or too large. In our experiments, the best classification performance is achieved when M=35. However, in order to ensure fairness when comparing with the baseline model in the comparative experiments, we set M=30 in the following experiments.

### 6.3. User Information and Sentiment Relationship Experiment

**Figure 4:**
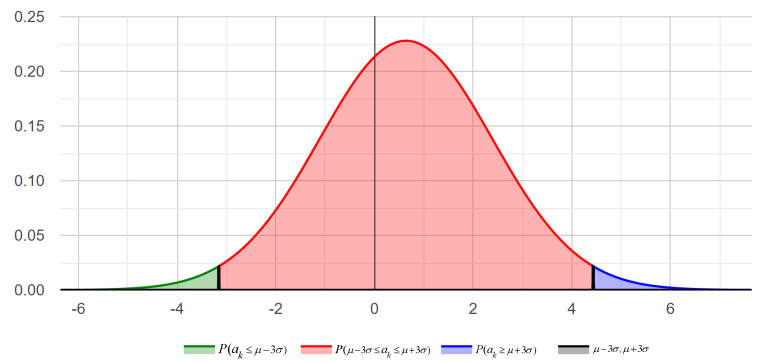
Yelp user sentiment normal distribution.

Figure 4 shows the sentiment distribution of the Yelp dataset, with a mean value μ of 0.64 and a standard deviation σ of 1.75. In the Yelp dataset, approximately 97% of the users are normal, while 3% of users have overly positive sentiments, as the sentiment values of biased users are several times higher than those of regular users. Therefore, the sentiment of these users may affect the judgment results for specific products, businesses, or topics.

According to our definition of biased users $BU_{k}$, we calculated the average sentiment scores to classify users into negative users and positive users. Additionally, we defined different sentiment score ranges concerning the mean value to display the intensity of the bias. Thus, the mean value serves as the metric for this study. In other words, our analysis of the correlation and relationship between user sentiment and selected user features is based on this metric. Table 5 shows the mean values for the dataset used in this study.

**Figure 5:**
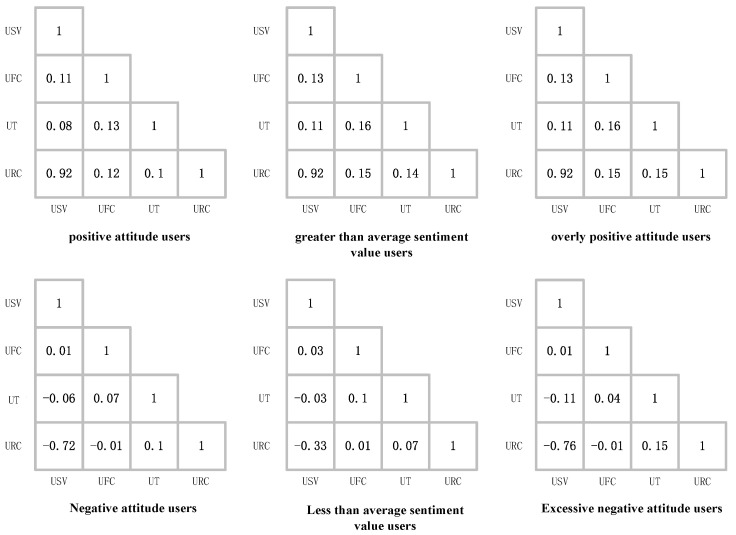
Results of calculating the correlation coefficient cr between “user review count (URC)”, “user tenure (UT)”, “user friends count (UFC)”, and “user sentiment value (USV)”.

As shown in Figure 5, there is a relationship between user features and sentiment values. Thus, a model can be built to identify biased users based on user features. We elaborate on this relationship below.

Based on the results of this study, which calculated the correlation coefficient cr of user features for the Yelp dataset used, the number of friends a user has within the network significantly affects their attitude. Figure 5 shows the results of calculating the correlation coefficient cr between the “user review count”, “user tenure”, “user friends count”, and “user sentiment value”. According to the results in Figure 5, we can see:

- The correlation coefficient cr value for the “friends count” and “sentiment value” of overly positive users is 0.13. These results indicate that for overly positive users, there is a weak positive correlation between the user’s “friends count” and “sentiment value”. The more friends a user has, the higher their tendency to form a positive attitude and the lower their tendency to form a negative attitude.
- The user’s tenure is related to their biased attitude. In the Yelp dataset, the correlation coefficient cr values of “tenure” and “sentiment value” for overly positive and overly negative users are 0.11 and −0.11, respectively. These results indicate that for overly positive users, there is a weak positive correlation between the user’s “tenure” and “sentiment value”. The longer they use Yelp, the higher their tendency to form a positive attitude and the lower their tendency to form a negative attitude. One explanation for these results is that people tend to have a more positive attitude towards a particular website the longer they stay on it, often because they enjoy the site.
- Interestingly, according to the correlation coefficient cr values of “review count” and “sentiment value” for overly positive and overly negative users, with cr values of 0.92 and −0.76, respectively, we found a strong positive relationship between “review count” and overly positive users, known as positive bias.

### 6.4. Comparative Experiment

We have selected a variety of traditional and recent methods for comparison in our sentiment classification task to assess the efficacy of the model proposed in this study.

- TextCNN [35]: Kim et al. use convolutional neural networks to extract text features, capturing key information in the text to help predict sentiment polarity. Furthermore, the TextCNN_CLIP model leverages CLIP to extract image features and combines them with the text representation to perform sentiment classification.
- FastText [36]: Bojanowski et al. suggest the incorporation of sub-word information in word representations. Despite its simple network architecture, it performs well in text classification tasks. We utilize it to generate word embedding representations for comparison with BERT.
- BiGRU [37]: Tang et al. employ gating mechanisms to overcome the challenge of modeling long-range dependencies in sequences, which in turn enhances the quality of text representations. BiGRU_CLIP also leverages CLIP for extracting image features and combines the resulting text and image feature representations for sentiment classification.
- HAN [33]: Yang et al. propose a hierarchical attention network that considers the importance of various words within a sentence and various sentences within a document before generating a document-level text representation. In order to classify the sentiment of reviews by combining text representation and image feature representation, HAN_CLIP adopts CLIP for image feature extraction and concatenates the resulting image feature representation with the text representation.
- BERT [32]: Devlin et al. proposed a pre-trained language model that can capture extreme long-range dependencies through multi-head attention. BERT is fine-tuned with the text content of the training set for sequence classification tasks.
- VistaNet [25]: Truong et al. propose a multimodal sentiment classification network based on HAN that leverages visual features to weight sentence representations.
- GAFN [38]: Du et al. utilize a gated attention mechanism to fuse textual and visual data, enabling them to leverage the benefits of multimodal information while minimizing the impact of noisy images.
- VisdaNet [29]: Our previously proposed model effectively utilizes multimodal information for knowledge expansion in short texts, visual distillation in long texts, and employs visual attention. It addresses the issues of feature sparsity and information scarcity in short text representations, filters out task-irrelevant noise information in long texts, and achieves cross-modal joint-level fusion.
- UsbVisdaNet: The model proposed in this paper makes effective use of user information and employs a user behavior attention mechanism to improve VisdaNet’s word encoder.

**Figure 6:**
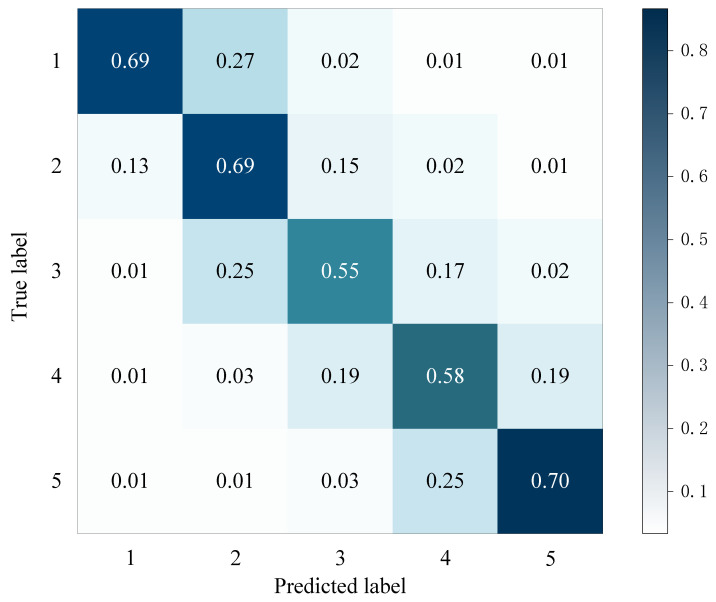
Confusion matrix for UsbVisdaNet on the test set.

We conducted experiments on the Yelp multimodal dataset to compare the performance of our proposed model with the sentiment classification baseline methods mentioned earlier. Accuracy was used as the evaluation metric. Table 6 displays the structural characteristics of each method. The experimental results, which are presented in Table 7, indicate that our proposed UsbVisdaNet model outperforms the other methods in terms of average accuracy. Our model achieved an accuracy that is about 5% higher than GAFN. Furthermore, our model outperformed the previous state-of-the-art (SOTA) model, GAFN, achieving the highest accuracy performance on the Los Angeles dataset, with an accuracy approximately 7% higher than that of GAFN. The model’s predictive performance on different ratings can be seen in the confusion matrix of UsbVisdaNet on the test dataset, which is presented in Figure 6.

It can be observed that our proposed model, UsbVisdaNet, achieves the best average accuracy performance on the Yelp multimodal dataset. Our model differs from the VistaNet model in that it includes a user behavior attention mechanism. The results of the comparative experiments support our hypothesis that user behavior is related to biased users, and the classification results affected by subjective biases in user reviews can be improved by utilizing user behavior information.

## 7. User Behavior Attention Visualization

In order to confirm our observations and substantiate the efficacy of user behavior attention, we illustrate some instances of reviews from the Yelp multimodal dataset, whereby we visualize the attention weights on a word-by-word level.

**Figure 7:**
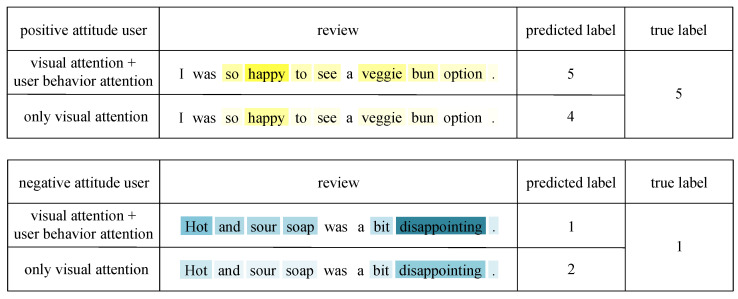
A sample of user behavior attention visualization. Blue depth represents negative emotional weighting, yellow depth represents positive emotional weighting.

The results are depicted in Figure 7, where it should be noted that the degree of color darkness correlates with the weight magnitude. For reviews written by users with a positive attitude, we observed that the word “happy” was assigned a higher attention weight after incorporating user behavior attention, resulting in a prediction closer to the true label. This may be because users with a positive attitude tend to give higher ratings even if the text of the review does not seem particularly favorable. Similarly, for reviews written by users with a negative attitude, we observed that the word “disappointing” was assigned a higher attention weight after incorporating user behavior attention, resulting in a prediction closer to the true label. This may be because users with a negative attitude tend to give lower ratings even if the text of the review does not seem particularly critical. Our model not only effectively captures the biased user features but also generates accurate predictions. The visualization of user behavior attention indicates that our model can identify positive and negative users and improve classification results by accounting for user bias through the incorporation of user behavior information.

## 8. Conclusions

Sentiment analysis and opinion mining research aim to develop methods for automatically extracting user opinions on products (such as movies and food) from their interactions on social networks. However, existing sentiment analysis methods struggle to distinguish the attitudes of different types of users, such as those who are consistently negative or consistently positive. This impacts the analysis of user reviews for businesses and products, thus necessitating a method to identify these two types of users. We propose a multimodal sentiment classification method based on user behavior attention networks, which assists in predicting the sentiment of biased user reviews by recognizing their psychological behavior. This method can identify both positive and negative users and can improve classification results affected by subjective biases in user reviews by utilizing user behavior information. After validation through ablation and comparative experiments, our method achieved 62.83% accuracy on the Yelp multimodal dataset, demonstrating the effectiveness of the user behavior attention mechanism.

## Figures and Tables

**Table 1:** User information contained in the Yelp dataset.

User Information	Notes
user_id	User ID
user_name	User’s name
user_review_count	Number of reviews written by the user
yelping_since	User’s join date on Yelp, converted to UNIX timestamp
user_useful	Number of useful votes received by the user
user_funny	Number of funny votes received by the user
user_cool	Number of cool votes received by the user
elite	A set of integers representing the years the user became elite
fans	Number of fans the user has
average_stars	Average rating for all of the user’s reviews
compliment_hot	Number of hot compliments received by the user
compliment_more	Number of more compliments received by the user
compliment_profile	Number of profile compliments received by the user
compliment_cute	Number of cute compliments received by the user
compliment_list	Number of list compliments received by the user
compliment_note	Number of note compliments received by the user
compliment_plain	Number of plain compliments received by the user
compliment_cool	Number of cool compliments received by the user
compliment_funny	Number of funny compliments received by the user
compliment_writer	Number of writer compliments received by the user
compliment_photos	Number of photo compliments received by the user

**Table 2:** Yelp multimodal dataset.

Datasets	City	#Reviews	Max. #s	Avg. #s	Min. #w	Max. #w	Avg. #w	#Images	Min. #Images
	BO	315	85	13.4	14	1099	211	1654	3
	CH	325	96	13.5	15	1095	208	1820	3
Test	LA	3730	104	14.4	12	1103	223	20,254	3
	NY	1715	95	13.4	14	1080	219	9467	3
	SF	570	98	14.8	10	1116	244	3243	3
Valid	-	2215	104	14.8	12	1145	226	11,851	3
Train	-	35,435	104	14.8	10	1134	225	196,280	3
Total	-	44,305	104	14.8	10	1145	237.3	244,569	3

**Table 3:** Hyperparameter settings.

Hyperparameters	Settings
batch size	32
*x*	3
learning rate	0.0001
optimizer	Adam
number of classes of prediction	5
dropout rate	0.5
*M* (number of sentences in each review)	30
*H* (number of images per review)	3
*T* (number of words in each sentence)	30
sentence representation dimension	512
image representation dimension	512
attention dimensions	100
GRU representation dimension	50
word representation dimension	512
bidirectional GRU representation dimension	100

**Table 4:** The impact of the hyperparameter *M* on the model’s classification performance (accuracy) and the required training time.

*M*	Boston	Chicago	Los Angeles	New York	San Francisco	Mean	Time Cost (s) *
20	64.64	66.66	61.31	62.32	59.28	61.82	2072
25	64.95	65.74	61.77	60.92	61.39	61.86	2508
30	63.17	64.65	**63.02**	**62.54**	61.21	62.83	2894
35	**65.99**	**67.89**	62.76	62.43	**61.39**	**62.96**	4061
40	58.61	64.39	61.15	60.74	59.38	60.83	4832

* Time cost shows how much time was spent on the training and validation sets in order to train the best model. The unit is seconds.

**Table 5:** Average sentiment values of users in Yelp dataset.

Dataset	Average User Sentiment	Average Sentiment of Positive Users	Average Sentiment of Negative Users
Yelp	0.64	0.76	−0.18

**Table 6:** Structure comparison with baseline methods.

Model	Textual Features	Visual Features	Hierarchical Structure	Visual Attention	VD & KA	User Behavioral Attention
**UsbVisdaNet**	✓	✓	✓	✓	✓	✓
VisdaNet	✓	✓	✓	✓	✓	-
GAFN	✓	✓	-	✓	-	-
VistaNet	✓	✓	✓	✓	-	-
BERT	✓	-	-	-	-	-
HAN_CLIP	✓	✓	✓	-	-	-
HAN	✓	-	✓	-	-	-
BiGRU_CLIP	✓	✓	-	-	-	-
BiGRU	✓	-	-	-	-	-
FastText	✓	-	-	-	-	-
TextCNN_CLIP	✓	✓	-	-	-	-
TextCNN	✓	-	-	-	-	-

**Table 7:** Comparison of classification performance (accuracy) of baseline models on the Yelp restaurant reviews dataset.

Model	Chicago	Los Angeles	Boston	San Francisco	New York	Mean
TextCNN	54.80	54.03	54.32	53.04	53.58	53.88
TextCNN_CLIP	55.45	54.36	55.61	53.47	54.16	54.34
FastText	59.38	55.49	61.27	55.44	56.15	56.12
BiGRU	56.02	56.45	54.94	52.80	58.27	56.52
BiGRU_CLIP	57.24	56.60	58.69	55.48	57.02	56.74
HAN	58.53	57.61	61.60	53.02	57.14	57.33
HAN_CLIP	62.15	58.45	62.22	58.95	59.77	59.19
BERT	60.71	59.17	60.13	60.24	58.89	59.31
VistaNet	63.08	59.95	63.17	59.65	58.72	59.91
GAFN	**66.20** *	59.00 *	61.60 *	60.70 *	61.00 *	60.10 *
VisdaNet	62.77	62.57	62.86	60.70	62.10	62.32
**UsbVisdaNet(ours)**	64.65	**63.02**	**63.17**	**61.21**	**62.54**	**62.83**

* denotes the data we obtained from the original paper published by the original authors.

## Data Availability

The datasets involved in this study are available in publicly accessible repositories. The Yelp dataset can be found at https://github.com/PreferredAI/vista-net, accessed on 24 November 2021.
